# Drying without senescence in resurrection plants

**DOI:** 10.3389/fpls.2014.00036

**Published:** 2014-02-12

**Authors:** Cara A. Griffiths, Donald F. Gaff, Alan D. Neale

**Affiliations:** School of Biological Sciences, Monash UniversityClayton, VIC, Australia

**Keywords:** desiccation tolerance, senescence, drought, *Sporobolus stapfianus*, photosynthesis

## Abstract

Research into extreme drought tolerance in resurrection plants using species such as *Craterostigma plantagineum*, *C. wilmsii*, *Xerophyta humilis*, *Tortula ruralis*, and *Sporobolus stapfianus* has provided some insight into the desiccation tolerance mechanisms utilized by these plants to allow them to persist under extremely adverse environmental conditions. Some of the mechanisms used to ensure cellular preservation during severe dehydration appear to be peculiar to resurrection plants. Apart from the ability to preserve vital cellular components during drying and rehydration, such mechanisms include the ability to down-regulate growth-related metabolism rapidly in response to changes in water availability, and the ability to inhibit dehydration-induced senescence programs enabling reconstitution of photosynthetic capacity quickly following a rainfall event. Extensive research on the molecular mechanism of leaf senescence in non-resurrection plants has revealed a multi-layered regulatory network operates to control programed cell death pathways. However, very little is known about the molecular mechanisms that resurrection plants employ to avoid undergoing drought-related senescence during the desiccation process. To survive desiccation, dehydration in the perennial resurrection grass *S. stapfianus* must proceed slowly over a period of 7 days or more. Leaves detached from the plant before 60% relative water content (RWC) is attained are desiccation-sensitive indicating that desiccation tolerance is conferred in vegetative tissue of *S. stapfianus* when the leaf RWC has declined to 60%. Whilst some older leaves remaining attached to the plant during dehydration will senesce, suggesting dehydration-induced senescence may be influenced by leaf age or the rate of dehydration in individual leaves, the majority of leaves do not senesce. Rather these leaves dehydrate to air-dryness and revive fully following rehydration. Hence it seems likely that there are genes expressed in younger leaf tissues of resurrection plants that enable suppression of drought-related senescence pathways. As very few studies have directly addressed this phenomenon, this review aims to discuss current literature surrounding the activation and suppression of senescence pathways and how these pathways may differ in resurrection plants.

## INTRODUCTION

In crop plants, drought-induced senescence can cause nutrient loss and limit the growth phase resulting in a substantial reduction in yield. A major goal of agricultural science is to increase food production in a water-limited environment. Delaying drought-induced senescence to allow retention of higher chlorophyll levels has considerable potential to increase crop production under water-deficit (Gan and Amasino, 1997; Jordi et al., 2000; Sykorová et al., 2008). An understanding of the mechanism whereby resurrection plants suppress drought-induced senescence may enhance our knowledge of an important biological process and provide a novel means of increasing crop yields under adverse conditions.

Much resurrection plant research has focused on understanding how these plants survive desiccation and how desiccation-tolerance pathways are regulated. Approaches have involved identifying genes that are differentially regulated during dehydration and more recently, global analyses of the transcriptome (Oliver et al., 2004; Rodriguez et al., 2010), proteome (Oliver et al., 2010) and metabolome (Oliver et al., 2011) in dehydrating and rehydrating tissue have been undertaken. This information, when coupled with the detailed molecular genetic information available on drought responses of non-resurrection plants, may allow a more targeted molecular approach to identifying those genes functioning in dehydration-related pathways which are thought to be pivotal to the ability of resurrection plants to survive extreme dehydration. Such genes could then be utilized to increase the stress-resilience of important food crops.

In many non-resurrection plants, surviving water-deficit involves the up-regulation of drought tolerance genes that alter the osmotic potential of plant tissues to enable water retention (Zhang et al., 1999). When water loss becomes more severe, non-resurrection plants initiate leaf senescence, which is thought to be an efficient strategy for surviving water-deficit by reducing canopy size and transpiration, and allowing remobilization of water and nutrients to preserve organs crucial for the future survival of the plant (Chaves et al., 2003; Munné-Bosch and Alegre, 2004). Although the perennial resurrection grass *Sporobolus stapfianus* initiates similar osmotic responses to slow down the rate of dehydration and allow the desiccation tolerance program to be established, the survival of *S. stapfianus* does not rely on the retention of water but rather on the ability of the plant to withstand complete desiccation (Gaff and Ellis, 1974). Hence the initiation of leaf senescence, as a vital strategy to preserve water and cellular nutrients for the remaining plant tissues, does not occur in resurrection plants.

This leads to the question of how resurrection plants suppress the drought-induced senescence response. It is also not clear if a similar senescence-suppression pathway occurs during seed development. In most plants, dry mature seeds, and pollen tissues for a certain period of time, are composed of desiccation-tolerant tissue capable of producing new life (Walters et al., 2005) and at least some resurrection plants are thought to have evolved their vegetative desiccation-tolerance pathways by ectopic expression of desiccation-related pathways normally found in reproductive tissue (Oliver et al., 2000). This suggests that many plants may contain the required desiccation tolerance genes within the genome (Bartels, 2005). Many desiccation-tolerance-associated genes have homologs in non-resurrection plants but they appear not to be activated in vegetative tissue during dehydration.

The ability of resurrection plants to preserve healthy tissues and completely avoid senescence during dehydration is a key feature of desiccation tolerance. By attempting to synthesize the current knowledge of the desiccation program in the resurrection grass *S. stapfianus* with that of age- and drought-related senescence in non-resurrection plants, we hope to shed some light on possible mechanisms associated with dehydration-related senescence avoidance in resurrection plants.

## LEAF PROCESSES IN DEHYDRATING *S. stapfianus*

The desiccation of resurrection plants involves whole plant dehydration to an air-dried state with minimal cellular damage. The leaves of the desiccation tolerant grass *S. stapfianus* can survive drying down in equilibrium with air at 2% relative humidity, and remain in this anabiotic state for 2 years (Gaff and Ellis, 1974). The leaf signal initiating the desiccation-tolerance program appears to originate from a decreased water supply in the roots of *S. stapfianus*, as dehydration of plants with disturbed roots can lead to death of the plants (Gaff et al., 1997). Investigations into cellular structure during the dehydration of *S. stapfianus* leaves have revealed that there is a higher incidence of closed stomata and epicuticular wax on the adaxial leaf surface. The closing of stomata reduces water loss and water flux throughout the plant and reduces the transpiring leaf area (Tardieu, 2005). These features may aid in the deceleration of cellular dehydration in order to protect thylakoid membranes and allow time for the induction of pathways that facilitate desiccation tolerance in *S. stapfianus* (Quartacci et al., 1997). In addition, large bulliform cells in leaf tissue have a reduced rate of water loss, suggesting that they may act as an internal water reservoir, again aiding in slowing the rate of tissue dehydration (Dalla Vecchia et al., 1998). It has been shown that a higher rate of photorespiratory electron transport occurs in desiccation sensitive leaves undergoing drought-induced senescence in comparison to desiccation-tolerant leaves. This suggests that the protection of desiccation-tolerant leaves is not aided by the capacity of photorespiration to scavenge free electrons (Martinelli et al., 2007).

*Sporobolus stapfianus* retains around 40% of chlorophyll content in dry leaves which reconstitutes quickly to around 90% on rehydration (Gaff and McGregor, 1979).

Resurrection plants have evolved strategies to reduce the mechanical stress of cell wall stiffening during water loss. Arabinose-rich polymers have been implicated in maintenance of cell wall flexibility in several resurrection plants (Moore et al., 2013). Various resurrection plants may also employ drought-inducible cell wall modifications including calcium ion deposition, xyloglucan remodeling, and elevated cell wall expansins which can act to increase cell wall flexibility and allow cells to contract and fold without collapsing (Quartacci et al., 1997; Wu et al., 2001; Jones and McQueen-Mason, 2004; Vicré et al., 2004). In *S. stapfianus* the increase in poly-unsaturated phospholipids which accumulates during dehydration is thought to increase cellular membrane fluidity (Quartacci et al., 1997). Maintenance of cellular volume during dehydration, via water replacement in vacuoles with substances such as sugars, proline, polyphenols, and glycerol can also occur (Vander Willigen et al., 2004; Moore et al., 2007; Farrant et al., 2009). In some cells of resurrection plants, the controlled fragmentation of the vacuole into multiple smaller vacuoles may act to facilitate mechanical stabilization (Quartacci et al., 1997; Vander Willigen et al., 2004).

The drought-induced growth retardation in *S. stapfianus* is thought to be rapid compared to plants that utilize well-developed drought avoidance mechanisms (Puliga et al., 1996). The accumulation of amino acids (Gaff and McGregor, 1979) and sugars, in particular sucrose (Ghasempour et al., 1998) is also apparent during dehydration in *S. stapfianus* leaves. The co-ordination of carbon partitioning between these competing pathways alters throughout dehydration. In the initial stages of dehydration both cellular starches and photosynthesis direct equal energy into both these pathways (Whittaker et al., 2007). In the later stages of dehydration, after photosynthesis ceases and the starch stores are exhausted, carbon flux is directed to sucrose and amino acid biosynthesis. However, this increase in amino acid may also be attributable to insoluble protein breakdown (Whittaker et al., 2007). Amino acid accumulation has been associated with the stabilization of cytoplasmic constituents, ion sequestration and water retention (reviewed in Chen and Murata, 2002). The increase in sucrose is associated with a process known as vitrification, where the formation of biological glasses in the drying cell protects organelles from damage. The interaction between accumulating sugars and dehydration-induced late embryogenesis abundant (LEA) proteins (Ingram and Bartels, 1996), which are commonly associated with embryo development at the later stages of seed maturation (Dure, 1993), is thought to be important for the protection of cellular components. Recently, sucrose and glucose has been shown to accumulate in all viable desiccation tolerant tissue (Martinelli, 2008). In the rehydrating resurrection plant *S. stapfianus*, the initial energy requirement for revival appears to be generated preferentially from catabolism of the accumulated amino acids (Whittaker et al., 2004).

## GENE EXPRESSION IN DEHYDRATING *S. stapfianus*

Changes in leaf gene expression during dehydration in *S. stapfianus* was first examined by Gaff et al. (1997) where mRNA was isolated from air dry, dehydrating and fully hydrated leaf tissue, and translated *in vitro*. This process revealed novel genes being expressed during dehydration. A comparison of *in vivo* protein extracts with the *in vitro* translated proteins suggested that some dehydration-related transcripts may not be translated until the rehydration stage. These products are presumably required to protect against cellular damage during the rapid rehydration which generally occurs within 24 h following rainfall (Gaff et al., 1997) or to allow for the very rapid growth that occurs during the short wet periods when conditions are favorable (Blomstedt et al., 2010).

Early work on isolating genes up-regulated during the initial stages of dehydration of *S. stapfianus* leaf tissue identified a number of genes encoding dehydrins, group 3 *LEA*, glyoxalase I, thiol proteases, an eukaryotic initiation factor 1A (eIF1A) protein translation initiation factor, glycine- and proline-rich proteins, a tonoplast intrinsic protein, and an early light inducible protein (Blomstedt et al., 1998a,b; Neale et al., 2000; Le et al., 2007). A more extensive list of *S. stapfianus* dehydration-responsive genes is available in Gaff et al. (2009). Many of these genes have high levels of homology to genes associated with protection from dehydration in many non-resurrection plants or are expressed in desiccated seeds of higher plants. Some of these proteins have been suggested to be involved in the rapid translation of proteins during dehydration, stabilization of membranes during desiccation, solute flow during dehydration/rehydration, and reducing photosynthetic apparatus damage (Neale et al., 2000). Interestingly these genes were identified as being differentially expressed in desiccation-tolerant tissue and not in tissues dehydrated following excision from the hydrated plant, or in the related *S. pyramidalis*, both of which are desiccation-sensitive (Gaff et al., 1997). This indicates that an elevated and/or persistent drought response occurs in desiccation-tolerant tissues. In some cases the desiccation-tolerance-related genes were specific sequences from multi-gene families. The significance of this is not well understood. In severely dehydrated or desiccated resurrection grass tissue, the profile of the transcript pool is somewhat different compared to that of early dehydration stages. As might be expected, several of the transcripts present in high levels in desiccated resurrection plant tissues encode growth-related protein products for utilization upon rehydration (Blomstedt et al., 2010; Islam et al., 2013).

While this research has uncovered some of the key genes being up-regulated during dehydration of *S. stapfianus* and has set up the framework for understanding the molecular basis of desiccation tolerance, we are some distance from identifying the pivotal molecular mechanisms responsible for the successful negotiation of a complete desiccation and rehydration cycle. By necessity one of these mechanisms must involve suppression of the dehydration-induced cell death response which is implemented by many higher plants.

## LEAF SENESCENCE

Leaf senescence is a complex, degenerative, developmentally regulated programed cell death (PCD) process that is associated with the final stages of leaf development. Both multiple developmental and environmental signals (drought, detachment, and darkness) can affect this process (Lim et al., 2003). Leaf senescence is thought to have evolved to allow remobilization of nutrients and other molecules to younger, actively growing areas of the plant (Nam, 1997). Generally, plants will exhibit two types of age-related senescence, one being replicative (mitotic) senescence (associated with the inability of the cell to continue cell division upon aging) and post-mitotic senescence (associated with the degenerative process occurring after cellular maturation, for example, leaf organ maturation; Lim et al., 2003). Stress-related senescence, most similar to post-mitotic senescence, is degenerative involving the dismantling of cellular organelles, degradation of protein, nucleic acid, lipid, and chlorophyll, and the remobilization of nutrients and nitrogen compounds (Smart, 1994). During the senescence process, cellular respiration is maintained and transcriptional machinery remains active, being required to transcribe senescence-associated genes (SAGs).

## AGE-DEPENDENT SENESCENCE

Age-related senescence in perennial plants occurs from the oldest to the youngest leaves (Munné-Bosch, 2008). In different desiccation-tolerant angiosperm plant species, the interaction between the developmental stage of individual leaves, drying and senescence varies widely. In *S. stapfianus*, the leaves that have more recently attained full length will survive desiccation entirely, whereas in older leaves, the tissue at the tip of the leaves dies. As the leaves age, more of the length of the leaf from the tip dies during dehydration (Gaff and Giess, 1986; Gaff, 1989; Martinelli et al., 2007). Two extremes of the interaction are found *inter alia* in species of *Borya* (a genus formerly in the Liliaceae). In *Borya scorpioides* Lindley only the basal 4 mm of the youngest three leaves recover from desiccation, whereas 20 successive leaves on a shoot of *Borya mirabilis* Churchill survive desiccation (Gaff and Oliver, 2013).

Rehydration of young *in vitro*-cultured *S. stapfianus* seedlings that have been allowed to desiccate, results in the revival of the entire plant. Senescence of the oldest healthy leaves in more mature soil-grown *S. stapfianus* plants (Martinelli et al., 2007) suggests that desiccation tolerance of leaf tissue has an age-specific component and genes associated with repression of drought-induced senescence are not expressed in older leaves. Regulation of replicative leaf senescence may be responsible for the senescence of older leaves in *S. stapfianus* during desiccation. As the senescence of older leaves in plants has been shown to aid in the remobilization of nutrients to younger tissues (Munné-Bosch and Peñuelas, 2003) it is possible that senescence of older tissues in dehydrating *S. stapfianus* provides resources in the younger leaves for survival of the desiccation/rehydration cycle.

Telomere length, a suggested notification of cellular age has been shown to control replicative senescence in mammals (reviewed by Campisi, 1997). However, the effect of changes in telomeres as a result of cell replication in plants is not well studied. The length of telomeres in *Arabidopsis thaliana* at different developmental stages has not been shown to change; suggesting age-related senescence is not triggered by a reduction in telomere length in plants. However, plant telomeric DNA is involved in a DNA–protein complex with a telomere binding protein ATBP1 (*A. thaliana* binding protein 1) which is bound to the telomeric DNA in all stages of development. During the onset of senescence, a protein–protein interaction between ATBP1 (bound to the telomeric DNA) and ATBP2 (*A. thaliana *binding protein 2) occurs. This binding of ATBP2 during the onset of replicative senescence suggests that it may be disturbing the DNA–ATBP1 complex, thereby inducing senescence (Zentgraf et al., 2000). The progression of the age-dependent senescence process may also be facilitated by lipid degrading enzymes that can provide precursors for the synthesis of the senescence-promoting hormone jasmonic acid (JA; He et al., 2002). In animals, a high metabolic rate associated with elevated oxidative stress may be a determining factor in aging (Guarente, 1997). As yet there is scant evidence for this phenomenon in plants, although the delayed senescence, which appears to be specifically age-related in the *Arabidopsis ore4-1* mutant, may be attributable to a partially impaired chloroplast function providing a reduced metabolic rate (Woo et al., 2002).

Live air-dry leaves are easily distinguished from dead air-dry leaves (senesced) by color differences on drying *S. stapfianus* plants and during analyses of gene expression associated with desiccation-tolerance the senescing tissue would normally be excluded. A comparison of gene expression occurring in live and senescing leaf tissue from the same dehydrating plant may be useful for distinguishing drought-induced senescence genes from drought-induced protective genes and could provide evidence that ATBP2 or other as yet unidentified genes are up-regulated by drought stress specifically to induce senescence.

## SENESCENCE-ASSOCIATED GENES

Research into senescence in several species such as rice, *Arabidopsis*, tomato, and maize has identified a large number of SAGs responsible for the execution of the senescence process (He and Gan, 2002). Up to 15% of *Arabidopsis* genes may be differentially regulated during senescence (Buchanan-Wollaston et al., 2003; Guo et al., 2004; Zentgraf et al., 2004). The expression patterns of SAGs up-regulated during leaf aging and the roles SAGs play during senescence have been reviewed in detail by Buchanan-Wollaston (2008). Basal expression of SAGs is observed across many stages of leaf development, indicating that the genes play a role in non-senescent tissue, and increase in expression as the leaf ages (Weaver et al., 1998). In *Brassica napus* genes identified as being associated with senescence encode two types of protease, glutamine synthase, ATP sulfurylase, two types of metallothionein, ferritin, catalase, and an antifungal protein (Buchanan-Wollaston and Ainsworth, 1997) as well as chitinase, and PR1 (pathogenesis-related 1; Guerrero et al., 1990). The action of proteases during senescence has been suggested to be involved in senescence-related protein degradation (Lohman et al., 1994) which is one of the first cellular responses to senescence induction. The remobilization of nutrients may be attributed to the action of glutamine synthase and metallothioneins, although metallothioneins may also provide a protective role by scavenging free ions. Further protection may be offered by catalase, which detoxifies oxygen radicals (Buchanan-Wollaston and Ainsworth, 1997). The roles of the antifungal and pathogenesis-related proteins during senescence are not understood, although chitinase may have a role in cell wall disruption and cell signaling affecting hormone homeostasis (Kasprzewska, 2003). The pathogenesis-related protein, PR10, has recently been shown to interact with leucine-rich repeat protein 1 to initiate cell death-mediated defense (Choi et al., 2003) suggesting that the function of some pathogenesis-related proteins may be associated with signaling induction of the cell death pathway. In *Arabidopsis*, a number of *YLS* (yellow leaf specific) genes were found to be up-regulated during natural senescence (Yoshida et al., 2001). Of these genes, a lipid transfer protein (*YLS*3) was shown to up-regulated in the early stages of leaf yellowing, and genes up-regulated during the later stages of senescence were homologous to β-glucosidase (*YLS1*), strictosidine synthase (*YLS*2), aspartate aminotransferase (*YLS*4), protease I (*YLS*5), cytochrome P450 (*YLS*6). Some of the *YLS* genes could play similar roles to the *Brassica napus* *SAGs* discussed previously. *YLS4* is thought to be involved in nutrient remobilization. The hypothesized role for *YLS1* is to supply glucose for respiration whilst *YLS3* may be involved in lipid transfer activity. The function of several of these *YLS* genes (*YLS2, -5, -6, -7, -8, -9*) remains unknown (Yoshida et al., 2001). Interestingly, in artificially induced senescence [by application of darkness, ethylene, or abscisic acid (ABA)] not all of these *YLS* genes were responsive. A subtractive hybridization technique identified a suite of *Arabidopsis* SAGs that were grouped in several categories involved in degradation and remobilization of metabolites, the production of antioxidant- and defense-related compounds, and secondary metabolite biosynthesis (Gepstein et al., 2003). Included were several genes suspected of being associated with regulation of the initiation and progression of senescence. More recently, transcriptome analyses have revealed differential expression of around 800 SAGs associated with the dramatic change in physiology accompanying the PCD process (Buchanan-Wollaston et al., 2005; van der Graaff et al., 2006).

## INDUCTION OF SENESCENCE

**FIGURE 1:**
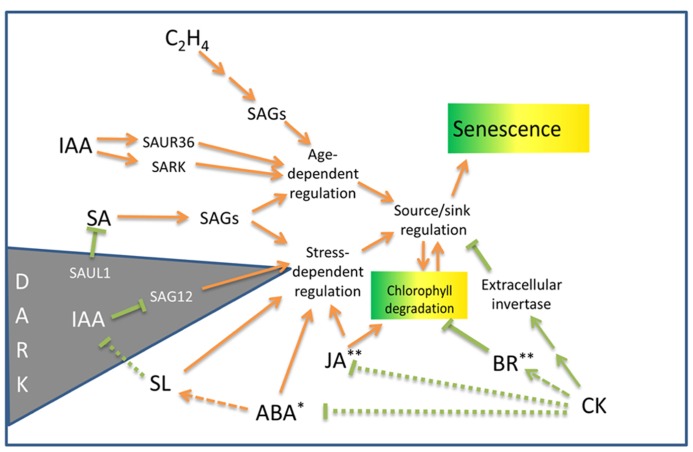
**Outline of hormonal influences on leaf senescence and chlorophyll degradation processes in non-resurrection plants.** SAGs, senescence-associated genes; SAUR, small auxin up RNA gene; SARK, senescence-associated receptor-like kinase; SAUL, senescence-associated E3 ubiquitin ligase (see text for details). Arrows indicate senescence promoting pathways. Lines terminated by bars indicate inhibitory pathways. Dotted lines indicate the mechanisms may be indirect. Asterisks indicate hormones that induce desiccation tolerance* or protoplasmic drought tolerance** in some resurrection plant species. For details on the genetic components associated with these pathways, see Fischer (2012) and Khan et al. (2013).

The regulation of PCD is vital to plant survival and requires the activity of several plant hormones to integrate a large number of external environmental influences in concert with the developmental stage of the plant (**Figure 1**). The actions of hormones can either facilitate or inhibit the senescence of leaf tissues. As yet, the network of regulatory mechanisms controlling SAG expression, and the precise molecular functions of many of these genes during senescence remains to be fully elucidated. The control and regulation of leaf senescence has been recently reviewed by Lim et al. (2007) and summarizes some of the genes thought to be involved in this process. In the early stages of leaf senescence, the activation of the SENESCENCE-ASSOCIATED RECEPTOR KINASE (SARK) may initiate the senescence program by functioning as a regulatory factor that perceives and transduces leaf senescence signals (Hajouj et al., 2000). Several other proteins involved in signal perception and transduction are also thought to play an early role in the induction of senescence and include the transcription factors WRKY53, a MYB protein and a zinc finger protein (Buchanan-Wollaston et al., 2003), as well as *AtSIRK* (*senescence-induced receptor-like kinase*) which is strongly expressed during leaf senescence (Robatzek and Somssich, 2002). Several WRKY genes have been found to regulate gene expression during pathogen defense and senescence, including some SAGs and *AtSIRK*, suggesting that the control or progression of senescence may rely on the action of these genes (Robatzek and Somssich, 2002; Zhou et al., 2011). Examples include WRKY70 which acts as a negative regulator of senescence during salicylic acid (SA), JA, or ethylene-mediated defense responses (Ülker et al., 2007). WRKY22 accelerates senescence but only under dark treatment (Zhou et al., 2011). WRKY53 also acts as a positive regulator during early senescence. Increasing levels of JA during senescence may activate the EPITHIOSPECIFYING SENESCENCE REGULATOR (ESR) protein which can bind to and inhibit the senescence-promoting activities of WRKY53 (Miao and Zentgraf, 2007). Hence WRKY transcription factors, which can form homo- and heterocomplexes (Xu et al., 2006) appear to act in a regulatory network integrating positive and negative influences on senescence (Miao and Zentgraf, 2007). Several members of the large family of NAC transcription factors found in plants also show differential expression during both age-related and dark-induced senescence and mutations in NACs have been shown to delay senescence (Lim et al., 2007). The E3 ubiquitin ligase, SENESCENCE-ASSOCIATED UBIQUITIN LIGASE 1 (SAUL1) acts as a negative regulator of senescence under low-light conditions via the targeting of senescence-promoting WRKY and NAC transcription factors. *Arabidopsis saul1* mutants placed in low-light conditions accumulate SA and exhibit chlorophyll loss and cell death (Raab et al., 2009; Vogelmann et al., 2012).

The hormone auxin (IAA) may have both positive and negative influences on senescence. Bioactive auxin increases twofold in senescing leaves and may play a positive role in senescence induction (Lim et al., 2007). Auxin induces the expression off the *SMALL AUXIN UP RNA* gene, *SAUR36*, as well as *SARK*, both of which promote senescence (Xu et al., 2011; Hou et al., 2013). Alternatively, overexpression of *YUCCA6* in *Arabidopsis* increases auxin levels and represses the senescence gene *SAG12*. Transgenic plants over-expressing *YUCCA6* are delayed in dark-induced senescence (Kim et al., 2011).

The induction of expression of several SAGs has been shown to be different during hormone and stress treatments. ABA and ethylene treatments increase the expression of many SAGs, but not necessarily to the same levels as do standard darkness-induced senescence assays. Hence, whilst there is overlap between natural- and stress-related senescence, a complex regulatory network operates to integrate the senescence process (Lim et al., 2003). The regulatory network has been reviewed by Fischer (2012) and Khan et al. (2013). Cytokinin, a noted inhibitor of senescence, reduces the expression of many SAGs (Weaver et al., 1998). Interestingly, dehydration produced a quick response, with half of the SAGs tested being up-regulated after 3 h of dehydration (Weaver et al., 1998). The results of this study indicates that the expression of some SAGs are associated with a general response to stress induced by both darkness and drought (Weaver et al., 1998) and provided some of the first data linking age-related SAG expression with drought.

Additional support for a common pathway shared between hormone-induced senescence and age-related senescence has been demonstrated by the effect of the mutation of the ORE9/MAX2 protein on senescence in *Arabidopsis* (Woo et al., 2001). ORE9, an F-box leucine-rich repeat signaling protein is expressed in the vasculature and plays multiple roles in different parts of the plant. ORE9/MAX2 mediates strigolactone signals produced in the roots in response to nutrient stress to suppress shoot branching (Stirnberg et al., 2007). Plants with defects in the strigolactone pathway exhibit delayed senescence (Snowden et al., 2005). This delayed senescence is thought to be mediated by reduced activity of ORE9/MAX2 which acts to promote leaf senescence and repress hypocotyl elongation in the light and is also involved in oxidative stress and drought responses (Woo et al., 2001, 2004; Stirnberg et al., 2002; Tang et al., 2005). Hormonally induced senescence utilizing ABA or ethylene or methyl jasmonate (MeJA), as well as age-induced senescence was delayed in *Arabidopsis* ORE9 mutants. This study suggests that ORE9 limits leaf longevity by removing proteins that delay leaf senescence via ubiquitin-dependent proteolysis (Woo et al., 2001).

Cytokinin has long been known to inhibit senescence (Richmond and Lang, 1957) and the delay of leaf senescence has been achieved by engineering the increased production of cytokinin in senescing tissues (Smart et al., 1991; Gan and Amasino, 1997). Increased levels of cytokinin during the initial stages of senescence inhibit degradation of chlorophyll and photosynthetic tissues (Smart et al., 1991). The protective effect of cytokinin on photosynthetic protein complexes appear to be mediated by repression of drought-induced ABA responses and by the upregulation of brassinosteroid synthesis and signaling pathways (Rivero et al., 2010). Xia et al. (2009) have shown that exogenous brassinosteroid can protect photosystem II (PSII) and promote photosynthesis. The activity of photosynthetic enzymes is also negatively regulated by accumulating sugar which can block the effects of cytokinin (Wingler et al., 1998). Correspondingly, the cytokinin-mediated delay of senescence is inhibited when extracellular invertase, the enzyme involved in sucrose cleavage and the phloem unloading pathway, is inhibited (Balibrea Lara et al., 2004). Although cytokinin has been shown to play a pivotal role in the progression of senescence, cytokinin deficit does not appear to trigger senescence onset. This was shown in a study by Werner et al. (2003), where cytokinin-deficient *Arabidopsis* did not display accelerated senescence. Similarly, an examination of the response of the *onset of leaf death 1* (*old1*) *Arabidopsis* mutant to ethylene exposure indicates that ethylene accelerates age-related senescence, but is not sufficient to initiate leaf senescence in younger plants (Jing et al., 2005). However, a number of genetic studies implicate JA and SA as well as ethylene in promoting senescence. Exogenous JA accelerates senescence in the leaves of *Arabidopsis* but is not effective in the JA-insensitive *coronatine insensitive 1* mutant (He et al., 2002). *Arabidopsis* plants defective in SA signaling exhibited delayed yellowing and reduced necrosis (Morris et al., 2000). However senescence appears to proceed fairly normally in plants deficient in JA, SA, and ethylene indicating that these hormones are not essential for initiating senescence (Grbić and Bleecker, 1995; Morris et al., 2000; Stintzi and Browse, 2000).

## CHLOROPHYLL DEGRADATION, PHOTOSYNTHESIS, AND SENESCENCE

The breakdown of the chloroplast occurs early during senescence. The accumulation of sugars and changes in cytokinin/ABA levels during drought stress results in degradation of chlorophyll, and a halt to cellular respiration (Richmond and Lang, 1957; Krapp et al., 1991; Smart, 1994). Chlorophyll breakdown during senescence is conducted by chlorophyll catabolic enzymes (CCEs) and begins with the removal of phytol and the Mg atom from chlorophyll, catalyzed by chlorophyllase and a metal chelating substance. The porphyrin cycle is then opened and pheophorbide *a* is removed by pheophorbide *a* oxygenase and red chlorophyll catabolite reductase. This releases a fluorescent catabolite which is exported from the plastid. The subsequent steps of chlorophyll breakdown involve converting the phytotoxic fluorescent breakdown products into non-fluorescent compounds, which are sequestered in the vacuole in a manner similar to widely occurring detoxification processes (Kreuz et al., 1996).

The regulation of chlorophyll degradation has been associated with the stay-green protein which binds to the light-harvesting complex II (LHCII) in the thylakoid membrane (Park et al., 2007). Recently LHCII has been shown to interact both directly and indirectly, with the five CCEs. Stay-green protein has been associated with the recruitment of CCEs in chloroplasts undergoing senescence leading to the breakdown of chlorophyll (Sakuraba et al., 2012). Staygreen mutants are defective in chlorophyll breakdown, however, the mechanisms that lead to this defect are not precisely known. The Staygreen mutants have the ability to retain chlorophyll during leaf senescence and are divided into types A–E. Briefly, type A delays the induction of senescence, but the rate of chlorophyll degradation is the same as the wild-type. Type B shows no delay in the onset of senescence, however, the degradation of chlorophyll and photosynthetic activity is much slower. Type C retains chlorophyll during leaf senescence, however, photosynthetic capabilities decrease. Type D results in the death of leaf tissue during drying or freezing and type E maintains a chlorophyll level higher than the wild-type throughout leaf development, however, the photosynthetic capacity of the plant does not increase (Thomas and Howarth, 2000).

Analysis of a type C Staygreen mutant has shown that stay-green protein acts upstream of pheophorbide *a* oxygenase in the chlorophyll breakdown pathway (Aubry et al., 2008). This Staygreen mutant has also been shown to regulate this process at a transcriptional level and that the ability of the plant to retain chlorophyll is associated with the failure to destabilize light-harvesting chlorophyll binding complexes in the thylakoid membrane, a prerequisite for chlorophyll degradation.

## CHLOROPHYLL DEGRADATION IN RESURRECTION PLANTS

Chlorophyll reduction/degradation is typically displayed in non-resurrection plants during prolonged dehydration as well as during senescence. Unlike many other resurrection plants, *S. stapfianus* only experiences partial chlorophyll loss during dehydration and thylakoid membranes are retained. Thus *S. stapfianus* is not considered either a poikilochlorophyllous (total chlorophyll loss and thylakoid degradation) or a homoiochlorophyllous (chlorophyll and thylakoid retention) resurrection plant (Gaff and McGregor, 1979; Quartacci et al., 1997). In *S. stapfianus* photosynthetic activity seems to be tightly coupled to water availability. Photosynthesis decreases gradually as the water content declines and does not cease till around 45% relative water content (RWC; Di Blasi et al., 1998). Following rehydration from the desiccated state, CO_2_ assimilation begins at 45% RWC and the chlorophyll content is rapidly restored. Hence *S. stapfianus* has a mechanism for preventing total chlorophyll degradation during desiccation. The ability of *S. stapfianus* to retain chlorophyll and reduce photosynthetic activity during dehydration has some similarities to the Staygreen type C mutant phenotype and an examination of the activity and regulation of pheophorbide *a* oxygenase in dehydrating *S. stapfianus* may be informative.

The LHCII/CAB transcripts in dehydrating *S. stapfianus* show a substantial increase at 60% RWC, before declining following further dehydration. Despite this reduction, the level of LHCII transcripts in dehydrating desiccation-tolerant leaf tissue remained higher than in the equivalent desiccation-sensitive leaf tissue (Blomstedt et al., 1998a). This is consistent with the maintenance of some photosynthetic activity in the dehydrating resurrection plant. Interestingly, Oliver et al. (2010) observed an increase in the abundance of type II light-harvesting LHCII during desiccation of *S. stapfianus*. The observation that LHCII in *S. stapfianus* increases during dehydration, despite the reduction in photosynthetic activity, suggests that the LHCII proteins may be stored for use in regaining photosynthetic capacity following rehydration. Alternatively, the increased abundance of LHCII may attenuate CCEs activity or recruitment by the stay-green protein and result in a reduction in the rate of chlorophyll breakdown during desiccation. An increase in the abundance of PSII stability/assembly factor during desiccation (Oliver et al., 2010), may also account for the protection of PSII. This factor is located in the lumen of stromal thylakoids and is essential for the formation of the PSII complex (Meurer et al., 1998). One or more of these mechanisms may operate to enable the plant to retain some photosynthetic activity during dehydration and to stabilize photosynthetic reaction centers in the air dried state so photosynthetic activity is regained rapidly after rehydration.

Some monocot and dicot species in Western Australia present an interesting case where foliage may become yellow during drought in summer or autumn and yet survive to regreen (in days or weeks) after winter rain (George, 2002). In most of such “diallagous” species (99 species, 59 genera, 24 families) there is no evidence that the foliage becomes air-dry; these may then represent an intermediate situation where drought results in the loss of chlorophyll, a process usually associated with senescence but here (as in most diallagous species) yellowing occurs without subsequent senescence and without induction of desiccation tolerance – an interesting situation that deserves investigation of the regulatory molecular mechanisms associated with it.

## ROLE OF CYSTEINE PROTEASE INHIBITORS (PHYTOSTATINS)

Cysteine proteases function in nitrogen remobilization during senescence and PCD (Grudkowska and Zagdańska, 2004) as well as modulating auxin physiology (Chen et al., 2007). Cysteine proteases may also be involved in degradation of LHCII (Forsberg et al., 2005). Transcripts encoding cysteine protease inhibitors (phytostatins) are up-regulated in dehydrating *S. stapfianus* (Blomstedt et al., 2010). The activity of cysteine protease inhibitors during dehydration may aid in the protection of chloroplast components, resource allocation, or may be decelerating drought-related senescence by inhibiting the breakdown of LHCII by cysteine proteases.

## CARBON FIXATION

A decrease in carbon fixation is associated with water deficits (Kramer, 1983). The primary enzyme involved in carbon fixation is ribulose-1,5-bisphosphate carboxylase/oxygenase (Rubisco). The synthesis of Rubisco is elevated during leaf expansion, whereas the rate of Rubisco synthesis declines during senescence and Rubisco is degraded (Suzuki et al., 2001). Between 15 and 30% of total nitrogen content in leaves is present in Rubisco. During senescence the Rubisco degradation products are used as a source of nitrogen in developing tissues (Makino et al., 1985). *S. stapfianus* displays a reduction in the abundance of Rubisco large subunit, as well as Rubisco small subunit binding proteins during desiccation (Oliver et al., 2010) similar to that displayed by senescing leaf tissues in desiccation-sensitive plants. In natural senescence, autophagy has been shown to be responsible for the degradation of Rubisco (Ono et al., 2013). Autophagic processes can be responsible for enveloping and remobilization of nutrients and maintenance of cellular viability under nutrient limited conditions. Furthermore, senescence is accelerated when autophagy is disrupted in *Arabidopsis* (Hanaoka et al., 2002). In rehydrating *S. stapfianus*, carbon fixation resumes at 40–45% RWC; initially rising rapidly and then more gradually to 75% of the level found in the initial hydrated plant (Di Blasi et al., 1998). *S. stapfianus* is able to resume normal metabolism 48 h after rehydration (Gaff and Ellis, 1974) suggesting the need for a pool of stored nutrients to achieving this state so rapidly after rehydration. Autophagy during dehydration in *S. stapfianus* may be responsible for the recruitment and relocation and storage of nutrients from older desiccation-sensitive leaves to younger desiccation-tolerant leaf tissues to enable rapid synthesis of carbon fixation enzymes and other proteins following rehydration. Interestingly, while the abundance of Rubisco declines, several other enzymes involved in carbon fixation increase in abundance during the dehydration of *S. stapfianus*. These enzymes include chloroplastic phosphoglycerate kinase, sedoheptulose 1,7-bisphosphatase, glyceraldehyde-3-phosphate dehydrogenase, chloroplastic aldolase, and phosphoribulose kinase. The accumulation of these enzymes suggests that a partial Calvin cycle may be required for the establishment of desiccation tolerance (Oliver et al., 2010). The increase in abundance of some of these enzymes which also have a role in glycolysis, may indicate a shift between autotrophy and heterotrophy during desiccation, and the degradation of Rubisco may supply nitrogen and other nutrients to the desiccating tissues to provide a nutrient source for the large increase in amino acids and sugars during desiccation (Whittaker et al., 2007). The application of exogenous sugar to mature spinach leaves results in a decrease in Rubisco and chlorophyll content, leading to a decrease in photosynthesis, whilst cellular respiration is stimulated (Krapp et al., 1991). This observation suggests some of the changes observed in desiccating *S. stapfianus* tissues may be driven by the accumulating sugar levels.

## ROLE OF SUGAR ACCUMULATION IN SENESCENCE

Cellular stability and vitrification within desiccating tissues of *S. stapfianus* has been attributed to sucrose accumulation (Ghasempour et al., 1998). The accumulation of sugars also occurs in drought-stressed tissues of non-resurrection plants and is thought to provide cellular protection during water-deficit (Wingler and Roitsch, 2008). Accumulation of glucose, fructose, and sucrose occur at the beginning of desiccation in *S. stapfianus*, and in the later stages of dehydration the glucose and fructose disappear and sucrose forms the leaf sugar pool (Ghasempour et al., 1998; Hoekstra et al., 2001). This large increase in the leaf sugar pool may not only protect the cellular constituents from damage but may also act as a signal to slow cellular metabolism once the cell is stabilized to withstand extreme dehydration. This theory is supported by the observation that an increase in sugar aids in the inhibition of photosynthesis (Krapp et al., 1991). In non-resurrection plants the presence of sugars in tissues is linked to the regulation of senescence during drought. In lettuce, increased cytokinin production during senescence results in an abnormally high accumulation of sugars in the upper leaves. This sugar accumulation causes premature senescence (McCabe et al., 2001). The causal relationship between sugar accumulation and the reduction of photosynthesis and initiation of senescence is complex. Research has shown that the senescence associate promoter P_SAG12_ is repressed by sugars, however, above a threshold level of sugar accumulation the expression of SAGs is induced (McCabe et al., 2001). High level sugar accumulation during dehydration is a common feature of resurrection plants and suggests that these plants have a mechanism that allows sugar to accumulate to high levels without triggering the induction of senescence.

## HORMONAL INVOLVEMENT IN DROUGHT-RELATED SENESCENCE AND ITS EVASION

The induction of senescence in the leaves of non-resurrection plants during adverse conditions has been the topic of several studies (Weaver et al., 1998; reviewed by Munné-Bosch and Alegre, 2004). Drought, darkness, pathogen attack, and oxidative stress can elicit senescence, remobilization of resources, and changes in growth patterns to diminish stress exposure (Potters et al., 2007). While there are differences in the genetic responses between stress-induced senescence and age-related senescence, there are also common elements that are mediated by cross-talk between hormones that trigger or repress senescence (Park et al., 1998; Weaver et al., 1998).

The relationship between senescence and cytokinin has long been recognized (Richmond and Lang, 1957) where increasing the endogenous cytokinin content can inhibit leaf senescence (McCabe et al., 2001; Rivero et al., 2010). It has been suggested that cytokinin mediates the source–sink relationship during age-related senescence (Roitsch and Ehness, 2000) with the source being the older leaves and the sink being the younger leaves. Interestingly, the production of cytokinins during senescence has also been associated with enhanced drought tolerance (Rivero et al., 2007). When cytokinin synthesis was enhanced using isopentenyltransferase (IPT) under the control of the SARK promoter in both tobacco and rice, drought-related senescence was reduced (Rivero et al., 2010; Peleg et al., 2011). The grain yield of the transgenic rice under drought stress was greater than the wild-type (Peleg et al., 2011). Hence, the focus of agricultural research into increasing crop yields through the inhibition of drought-induced senescence has for the most part been directed at increasing production of cytokinin via transgenic means. This increase in yield caused by the increase in cytokinin synthesis was also associated with the induction of brassinosteroid-associated gene expression, and a reduction in jasmonate-associated gene expression during water stress (Peleg et al., 2011). These studies demonstrate the complexity of hormone interaction associated with the drought-related senescence process.

## CYTOKININ ACTIVITY IN RESURRECTION PLANTS

Cytokinin levels have not been examined during dehydration of *S. stapfianus*, however, in the resurrection plant *Craterostigma wilmsii*, cytokinin concentrations during the initial stages of desiccation are low (Vicré et al., 2004). This low cytokinin content during the early stages of desiccation is similar to the situation that occurs during senescence in tobacco which is accompanied by a decrease in leaf cytokinin content (Balibrea Lara et al., 2004). However, a substantial increase in cytokinin occurs when *C. wilmsii* plants dehydrate below 20% RWC. During rehydration from the desiccated state, the cytokinin levels progressively decrease again and are reduced to initial low levels at 70% RWC (Vicré et al., 2004). A non-resurrection plant experiencing 20% RWC would be severely stressed and it seems unlikely that the increase in cytokinin content in *C. wilmsii* at 20% RWC and below would be a mechanism associated with halting senescence. It is not clear whether increased cytokinin is essential to allow the leaf tissue to survive the desiccated state or is associated with resumption of metabolism or regrowth after rehydration.

The cytokinin-mediated delay of senescence has been shown to involve extracellular invertase activity in tobacco (Balibrea Lara et al., 2004). Extracellular invertase contributes to the ability of plant cells to import sugars and undertake heterotrophic growth (Roitsch et al., 2003). In *Corallium rubrum*, the induction of extracellular invertase by cytokinin also induces a hexose transporter, which doubles sucrose accumulation (Ehness and Roitsch, 1997). If this is mirrored in resurrection plants, this could be a mechanism of accumulating sucrose in desiccating tissues for cellular protection and potential energy supply. Extracellular invertase and hexose transporter induction by cytokinin has not been studied in *S. stapfianus.* However, hexokinase activity, which regulates the entry of hexose sugars for primary metabolism and sucrose storage is associated with desiccation of both *S. stapfianus* and *X. viscosa* resurrection plants. Hexokinase activity peaks at 30% RWC, coinciding with the decline in cellular glucose and fructose content and rapid accumulation of sucrose beginning to occur at 50% RWC (Whittaker et al., 2001). Peak sucrose content in *S. stapfianus* occurs at 30% RWC and remains at a similar level below 30% RWC when the plant is considered air-dry (Whittaker et al., 2001). In non-resurrection plants a decrease in cytokinin levels may be required to allow senescence to proceed. The presence of cytokinin can block some cellular responses to sugar accumulation, which includes necrosis and chlorosis (Jang et al., 1997) as well as inhibiting chlorophyll and photosynthetic tissue degradation (Richmond and Lang, 1957). Sucrose accumulation in *S. stapfianus* continues essentially through the entire drying process and reaches levels around 10 times those found in hydrated plants (Gaff et al., 2009). This level of sugar accumulation is thought to be vital for surviving the final stages of desiccation where dehydration is extreme. As well as inhibiting photosynthesis, and thereby preventing potential damage generated by reactive oxygen species (ROS) and other toxic by-products arising from photosynthesis, the vitrification of cellular components by accumulated sugar allows this state to be sustained until rehydration without decomposition. Cytokinin increases, as seen in the severely dehydrated resurrection plant *C. wilmsii*, may aid the sugar accumulation process in the final stages of desiccation.

## ABA INTERACTIONS WITH CYTOKININ AND SUGAR ACCUMULATION

Abscisic acid has been implicated in the senescence of tissues during drought stress. In rice plants, ABA was shown to aid in carbon remobilization from senescing tissue into grain production (Yang et al., 2003). ABA has traditionally been thought of as mediating drought responses in many plants. An increase in endogenous ABA levels in tissues, leads to an increased transcription of stress-responsive genes which aids the plant in surviving drought stress (Sharp, 2002). In tomato, increasing endogenous levels of ABA within tissues has been demonstrated to enhance drought tolerance (Thompson et al., 2007). Hence ABA appears to have both cellular protection activities and senescence promoting activities. The precise nature of the interaction between ABA and cytokinin during drought stress is still under examination (Yamaguchi-Shinozaki and Shinozaki, 2006). It has been shown that increased cytokinin synthesis under the control of the SARK promoter in tobacco, prevented the activation of some drought-related ABA responses, including preventing the degradation of protein complexes involved in photosynthesis. Despite these reduced ABA responses, the increased cytokinin synthesis during drought stress produced a plant that is more drought tolerant than wild-type plants (Rivero et al., 2010). The transgenic plants survived drought for 15 days and retained higher photosynthetic activity and water content in comparison to the wild-type plants which did not survive under these conditions (Rivero et al., 2007). This demonstrates the antagonistic nature of plant responses to cytokinin and ABA and could be related to the opposing effects these two hormones have on growth signaling. Interestingly, the effect of ABA on growth signaling is observed in *Arabidopsis* mutants deficient in ABA synthesis or ABA signaling. These plants show no growth arrest when exposed to high sugar concentrations (reviewed by Rolland et al., 2006).

Whilst sugar accumulation during drought stress is thought to have a protective effect on cellular components and to delay water loss, sugar accumulation, along with ABA, may also act as a growth retardation signal via regulation of the growth inhibiting activities of Snf1-related protein kinase 1 (SnRK1; Jossier et al., 2009). SnRK1 has been strongly implicated in the control of metabolic enzymes and along with SnRK2 and SnRK3 acts in a network to link metabolic and stress signaling in plants (Halford and Hey, 2009). The activities of the tobacco SnRK1 complex can lead to reallocation of carbon resources (Schwachtje et al., 2006) and interestingly, in animal systems the “upstream” kinase (LKB1) of the SnRK1-related protein (AMPK) can trigger apoptosis in response to energy stress (Shaw et al., 2004). Sugar accumulation has also been suggested to be involved in redox signaling (Hare et al., 1998), as has SnRK1 (Halford and Hey, 2009). The environmental signals that affect metabolism via the SnRK family, including drought, cold, salt, and nutrient stress all impose sink-limiting growth conditions and may lead to sugar accumulation and ultimately cause senescence. Sink-limiting conditions refers to situations where the growth of tissues is impeded by adverse environmental conditions despite the ample supply of sugar. Nunes et al. (2013) have shown that trehalose-6-phosphate (T6P) accumulation correlates closely with sucrose accumulation under sink-limited conditions imposed by cold temperatures and N-limitation. This finding, along with the observation that T6P inhibits SnRK1 in growing tissues of plants (Zhang et al., 2009; Debast et al., 2011) has led to the proposal that whilst SnRK1 inhibits growth under conditions when sugar and energy is scarce, under sink-limiting conditions, T6P accumulates to inhibit the growth-retarding activities of SnRK1. This prepares the plant for rapid growth recovery in the presence of high sucrose following alleviation of the environmental stress (Nunes et al., 2013). In these experiments, alleviation of a cold-treatment produced a threefold higher growth rate than that of unstressed plants, which was dependent on T6P levels. A similar accumulation of growth-related transcripts and a growth rate increase is seen in *S. stapfianus* that has gone through a dehydration/rehydration cycle compared with plants kept fully hydrated (Blomstedt et al., 2010). T6P levels have not been measured in dehydrating *S. stapfianus* but trehalose increases up to 10-fold in *S. stapfianus* dehydrated below 40% RWC. However, the accumulated amount is too small to provide substantial protection alone (Gaff et al., 2009) and may be more consistent with a role in signaling. It is not clear that there is a direct link between trehalose accumulation and senescence inhibition, although senescence has been shown to be delayed in cut Gladiolus spikes by the immersion of the spike in trehalose, which increased the vase-life by 2 days (Otsubo and Iwaya-Inoue, 2000).

## REACTIVE OXYGEN SPECIES

Injurious ROS such as superoxide, peroxide, and free hydroxyl ions has been shown to increase during drought stress (Alscher et al., 2002) and the accumulation of ROS has been implicated in PCD (Del Río et al., 1998). Resurrection plants may experience high levels of irradiation when dehydrating in the hot sun, with the potential to incur photo-oxidative damage from the formation of ROS. Some desiccation-tolerant plants reduce photo-oxidative stress by losing all chlorophyll during drying (Tuba et al., 1993; Gaff et al., 2009). While *S. stapfianus* leaves retain most of its chlorophyll, the plant has the ability to produce protective pigments and decrease photosynthetic activity in synchrony with decreasing water availability. During drought stress, plants produce enzymes such as peroxidase (POD) and superoxide dismutase (SOD) to protect against damage to cellular components by ROS. These two enzymes are part of the first line of defense against ROS. In *S. stapfianus*, the Cu–Zn SOD which provides cellular protection in chloroplasts and cytosol decreases in abundance during dehydration. This is presumably due to the reduction of activity of SOD required, due to stabilization of cellular structures by vitrification (Veljovic-Jovanovic et al., 2006; Oliver et al., 2010).

Catalase transcripts in *S. stapfianus* increase initially during dehydration and peak during the mid-stages of drying (59–40% RWC), then drop to levels below those observed in fully hydrated plants as the tissue dries further (Blomstedt et al., 1998a). Upregulation of catalase activity, along with the activities of other antioxidant enzymes, has been demonstrated in several other resurrection plants during the onset of dehydration. Interestingly, these antioxidant enzymes were found to be substantially more resistant to dehydration-related damage than those from the non-resurrection plants examined (Farrant et al., 2007). The changes in catalase transcripts observed during dehydration in *S. stapfianus*, may be associated with the maintenance of photosynthetic activity during the early stages of drying, as catalase is responsible for the breakdown of hydrogen peroxide and provides protection against oxidative damage during photosynthesis (Kaiser, 1976; Forti and Gerola, 1977).

In one of the few studies undertaken on senescence in resurrection plants, Veljovic-Jovanovic et al. (2006) found that the isoforms of POD produced by the resurrection plant *Ramonda serbica* during senescence and dehydration are different. It was suggested that these isoforms may have alternate physiological roles, with a cationic isoform of POD being expressed during dehydration of *R. serbica*, to protect cellular constituents, and an anionic isoform involved in lignifying cell walls during senescence, presumably to impede pathogens transgressing the cell wall barrier.

## PROTEIN SYNTHESIS

During the dehydration-induced instigation of desiccation-tolerance pathways in resurrection plants it is important that cellular machinery remains active. Similarly, at least during the initial stages of drought-induced senescence, the activity of the transcriptional and translational machinery is maintained as the cell produces the required SAG products. However, desiccation-sensitive plant species cease protein synthesis at mild levels of drought stress; while desiccation tolerant species have the ability to continue protein synthesis until leaves are almost air-dry (Bartels et al., 1990; Gaff et al., 1997). Protein turnover during drying in resurrection plants is also essential for redirecting protein synthesis into production of protectant proteins. Increases in transcripts encoding proteases and protein translation factors during dehydration in *S. stapfianus* and may contribute to continuing protein turnover (Blomstedt et al., 1998b).

The content of soluble protein doubles in *S. stapfianus* leaves drying on intact plants as they became desiccation-tolerant, but not in desiccation-sensitive leaves dried detached (Whittaker et al., 2004). During rehydration, the soluble protein content in the desiccation-tolerant leaves reduces again to control levels. This suggests that the activity of the translation machinery is elevated during the drying down of resurrection plants.

There is some evidence that changes in expression levels, or production of particular isoforms, of protein turnover enzymes and translation machinery components may be important during dehydration of resurrection plants. Eukaryotic translation initiation factor 5A (eIF5A) has been shown to be implicated in plant growth and development (Chou et al., 2004; Thompson et al., 2004). eIF5A is involved in facilitating protein synthesis, however, the precise cellular function is not understood. Recently it has been shown that the abiotic stress responsive WRKY and RAV proteins, involved in the ABA signaling pathway, can bind to the promoter and drive expression of eIF5A. Yeast and poplar expressing eIF5A display elevated protein content, and an improved tolerance to abiotic stresses (Wang et al., 2012). Furthermore, separate isoforms of eIF5A facilitate the translation of mRNAs encoding proteins involved in cell division and cell death, suggesting a role of eIF5A in senescence (Thompson et al., 2004). Although expression of eIF5A has not been studied in *S. stapfianus*, an increase in the levels of eIF1A has been observed in dehydrating tissues (Neale et al., 2000). The role of eIF1A has been shown to be important in the tolerance to salt stress (Rausell et al., 2003), however, its role in dehydration stress and senescence has not been studied.

*Sporobolus stapfianus* may regulate the expression of eIF1A to allow the plant to facilitate dehydration-related protein synthesis during desiccation (Neale et al., 2000). In addition to translation initiation factors, *S. stapfianus* also has increased expression of elongation factors during dehydration. In *S. stapfianus*, eEF1A (eukaryotic elongation factor 1A) is expressed in dehydrating tissues with high levels of expression appearing in fully hydrated leaf tissue, at the initiation of the desiccation tolerance pathway at 60% RWC, and again at 20% RWC (Blomstedt et al., 2010). This elongation factor catalyses the binding of aminoacyl-tRNA to the A-site of the ribosome (Ejiri, 2002) demonstrating its ability to facilitate protein synthesis. Like eIF5A, eEF1A transcripts accumulate during abiotic stresses (Dunn et al., 1993). The function of eEF1A is not well understood, but it may allow for the rapid synthesis of proteins involved in avoiding the senescence program when the desiccation-tolerance program is induced (~60% RWC) and where sugar has accumulated to very high levels (~20% RWC).

## ENDOPLASMIC RETICULUM STRESS

**FIGURE 2:**
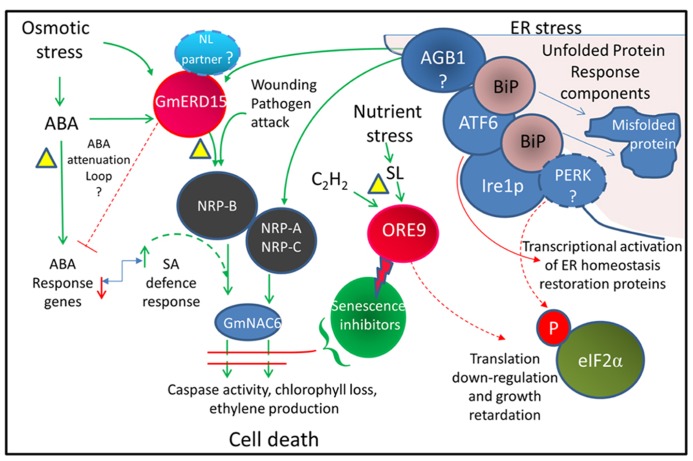
**Integrated cell death pathway model linking drought stress and ER stress.** Model showing pathways thought to be associated with activating cell death. Dotted lines represent proposed pathways. The triangles indicate possible drought-induced senescence inhibition points. The bent arrow indicates an antagonistic relationship. The lightning bolt indicates protein degradation. The model is based on the results of Alves et al. (2011) from *Glycine max* and also incorporates information from *Arabidopsis* studies. The ABA attenuation loop is based on AtERD15 (Kariola et al., 2006) which does not appear to activate NRP-B expression. The involvement of GmERD15, which contains a PAM2 domain, in the regulation of ABA signaling is yet to be demonstrated. AGB1, a heterotrimeric G protein thought to have a role in triggering the cell death signal; ATF6, activating transcription factor 6 related homologs (e.g., bZIP28 and 60); BiP, ER chaperone binding protein; Ire1p, inositol-requiring protein-1 homolog; NL, nuclear localization; NRP, N-rich proteins; PERK, protein kinase RNA-like ER kinase; SA, salicylic acid; eIF2α, eukaryotic initiation factor 2α (related references: Tang et al., 2005; Costa et al., 2008; Liu and Howell, 2010; Deng et al., 2011; Moreno and Orellana, 2011).

Endoplasmic reticulum (ER) stress has been linked with osmotic stress-induced cell death (Alves et al., 2011) where integrated signals from both ER stress and osmotic stress are required to activate cell death (**Figure 2**). In all eukaryote cells, including plants, ER stress triggers the unfolded protein response (UPR). The ER located molecular chaperone binding protein BiP assists in folding newly synthesized proteins in the ER lumen and also acts as a sensor of ER stress and stress-related signal generation (Reis et al., 2011). Environmental stress can lead to an accumulation of unfolded proteins in the ER. It is thought that a high level of unfolded proteins sequesters BiP, releasing and activating several ER-associated receptors such as PERK (protein kinase RNA-like ER kinase), IRE1 (inositol-requiring protein-1), and ATF6 (activating transcription factor-6). Activation of these receptors triggers the UPR, leading to a down-regulation of translation and an increase in ER protein folding and process components including BiP (Reis and Fontes, 2012). Several key components of the plant UPR have been identified although the precise roles of some of these components remain to be clarified (Fanata et al., 2013). Whilst no homolog of PERK has been identified in plants, GCN2 may play a similar role in phosphorylating eIF2α to inhibit protein synthesis (Zhang et al., 2003). bZIP60 acts as a plant homolog of ATF6 and up-regulates the BiP chaperone as well as SKP1, a component of the SCF-type E3 ubiquitin ligase complex, that degrades misfolded proteins via the 26S proteasome (Ye et al., 2011). When the stress is severe enough to prevent restoration of ER homeostasis, a signal is activated that involves activation of asparagine-rich protein (*NRP*) genes (Irsigler et al., 2007) and other downstream components that result in cell death (Costa et al., 2008; Wang et al., 2008). NRP expression causes chlorophyll loss, ethylene production, and senescence (Reis and Fontes, 2012). The transcription factor early responsive to dehydration 15 (ERD15; Kiyosue et al., 1994) has been identified in several plant species. ERD15 in soybean binds to the promoter and drives expression of the NRP-B gene and is proposed to link osmotic stress and ER stress to cell death (Alves et al., 2011). There may be differences in the roles of ERD15 from *Arabidopsis* and soybean. Both the *Arabidopsis* and soybean ERD15 protein contain PAM2 domains. PAM2 domains bind PABP and may be involved in regulating mRNA translation. ERD15 has been proposed to attenuate ABA signaling in *A. thaliana* lines, with silenced *ERD15* expression showing hypersensitivity to the exogenous application of ABA during seed germination, and the over-expression of *ERD15* resulting in tolerance to exogenous ABA (Kariola et al., 2006). In *Arabidopsis*, ERD15 does not appear to directly drive NRP-B expression but may act through attenuation of the ABA antagonistic effect on SA signaling, since the SA hypersensitive response appears to induce NRP expression (Ludwig and Tenhaken, 2001).

## SENESCENCE ATTENUATION MECHANISMS IN RESURRECTION PLANTS

By necessity resurrection plants must contain a mechanism for preventing activation of the UPR cell death response during severe dehydration. As resurrection plants can undergo age-related senescence, it would seem likely that the mechanisms for inhibiting drought-related senescence would operate upstream of the terminal stages of the PCD process (see **Figure 2**). This may involve attenuation of the activity of ERD15. An examination of the activity of ERD15 and potential interacting proteins during dehydration in resurrection plants may reveal if the UPR and cell death pathway is modified in these plants. As ERD15 is a focal point for integration of these two pathways, attenuation of ERD15 activity would allow the plant to withstand increased endogenous ABA levels and would also modulate ER stress signaling during dehydration to prevent induction of senescence in the younger desiccation-tolerant leaves.

Exogenous ABA induces full desiccation tolerance in hydrated leaves of the monocot *Borya constricta* (Gaff and Loveys, 1984). In the resurrection plant *C. plantagineum*, the induction of the desiccation tolerance pathway is also ABA-dependent (Bartels and Salamini, 2001). As *Borya constricta* is poikilochlorophyllous (Gaff, 1989) and *C. plantagineum* is homiochlorophyllous (Farrant et al., 2007) the importance of ABA in conferring desiccation tolerance cannot be inferred from the degree of chlorophyll exhibited by resurrection plants during dehydration. A non-ABA pathway appears to regulate desiccation tolerance in *S. stapfianus*. ABA in dehydrating *S. stapfianus* does not elevate substantially until the latter stages of dehydration, reaching a peak between 40 and 15% RWC. This is well after the initiation of the desiccation tolerance program which occurs around 60% RWC (Gaff and Loveys, 1993). While ABA accumulation in non-resurrection plants induces mechanisms that allow the survival of mild water-deficit, it can also induce chlorophyll degradation and senescence (Back and Richmond, 1971; Lim et al., 2003). In many desiccation-tolerant poikilochlorophyllous monocots, the link between dehydration-dependent chlorophyll degradation and leaf senescence has been uncoupled. Whereas in ABA-dependent homiochlorophyllous resurrection plants, such as *C. plantagineum*, it appears that a mechanism for attenuating both ABA-induced chlorophyll degradation and senescence as it dehydrates is present. An interesting function associated with ABA signaling has been proposed for the lysine-rich LEA-like protein CDeT11-24 from *C. plantagineum* based on its ability to bind phosphatidic acid (PA) *in vitro* (Petersen et al., 2012). LEA proteins, which are highly expressed in the leaves of resurrection plants during dehydration, have no apparent inherent catalytic activity but their predicted functions include the formation of stabilizing filaments within the cytoplasm and acting as molecular chaperones by retaining the hydration shell for proteins during desiccation (Wise and Tunnacliffe, 2004; Bartels, 2005). The *C. plantagineum* LEA-like protein, CDeT11-24 binds specifically to PA. PA is produced in plants in response to several stresses including drought and acts as a stress signal (Testerink and Munnik, 2005). A signaling function of PA involves the disruption of binding of 2C protein phosphatases (PP2Cs) to the ABA receptor (Park et al., 2009). PP2Cs, such as ABI1, act as negative regulators of ABA signaling and this disruption of receptor binding allows transduction of the ABA signal. Petersen et al. (2012) have hypothesized that the drought-induced membrane-binding of this lysine-rich LEA protein may interfere with the PA–ABI1 interaction and attenuate ABA signaling. Several LEA proteins that are differentially expressed in desiccation-tolerant tissue of *S. stapfianus* during dehydration have been identified (Blomstedt et al., 1998a,b; Le et al., 2007). Interestingly, one of the *S. stapfianus* lysine-rich LEA proteins, when ectopically expressed in *Arabidopsis*, locates to the chloroplast and appears to reduce ABA-related stress responses of the transgenic plants and to affect ABA-induced stomatal closure (Ling, 2011).

The activity of stress-related hormones, such as ABA, responsible for driving drought responses would normally be considered advantageous in the tissues of a dehydrating plant (Thompson et al., 2007). However, the rate of water-loss and growth retardation of resurrection plants under drought is rapid, when compared to many other plant species. This suggests that the primary survival response of resurrection plants like *S. stapfianus* experiencing drought is not to initiate measures to restrict water-loss or maintain normal metabolism, but to instigate the desiccation tolerance pathway. Hence, *S. stapfianus* may have several mechanisms to eliminate traditional stress-related hormonal responses to water-deficit during establishment of the desiccation tolerance program.

Apart from ethylene, the bioactivity of most known plant hormones can be modulated by the action of UDP-glycosyltransferases (UGTs; Lim and Bowles, 2004). The *S*porobolus *d*rought-responsive gene 8i, *SDG8i*, gene encodes a UGT whose transcript levels increase substantially under severe water deficit (Le et al., 2007). Whilst the endogenous substrate of this UGT is unknown, the enzyme has *in vitro* activity against the synthetic strigolactone analog GR24, suggesting that it may affect strigolactone signaling in the resurrection plant (Islam et al., 2013). Strigolactones are produced in the roots of plants in response to nutrient stress and translocate to the shoot to reduce shoot growth (Gomez-Roldan et al., 2008; Umehara et al., 2010). Strigolactones have pleiotropic effects on plant growth. They can regulate root growth in response to nutrient availability, affect flowering time as well as regulating shoot branching (Stirnberg et al., 2002; Dun et al., 2009; Leyser, 2009; Umehara et al., 2010; Ruyter-Spira et al., 2011). Mutations in the strigolactone synthesis and signaling pathways can also inhibit senescence (Woo et al., 2001; Snowden et al., 2005). When the *SDG8i* UGT is constitutively expressed in *Arabidopsis* it enhances the growth rate of the plant considerably, under both favorable and stress conditions. Auxin synthesis also appears to be upregulated in these plants. The constitutive expression of *SDG8i* UGT also delays senescence substantially (Islam et al., 2013). The *SDG8i* transgenic plants have a phenotype consistent with altered ORE9/MAX2 activity which acts as a signaling protein in the strigolactone pathway and promotes senescence (Woo et al., 2001; Stirnberg et al., 2007). While the effect on MeJA- and ethylene-induced senescence was not tested, *SDG8i* UGT activity was demonstrated to inhibit both dark-induced senescence and ABA-induced senescence (Islam et al., 2013). Low levels of the UGT transcript are present in hydrated leaf tissue in *S. stapfianus*. UGT transcripts increase substantially during dehydration and persist in desiccated tissue (Le et al., 2007). These results raise the possibility that the drought-induced expression of this UGT in *S. stapfianus* inhibits drought-related ABA-induced senescence and also promotes rapid plant growth following rehydration.

Enhanced disease resistance I (EDR1) encodes a protein kinase that acts as a negative regulator of ethylene- and SA-related senescence pathways in *Arabidopsis*. The ethylene pathway that EDR1 inhibits also appears to act through the reduced activity of ORE9/MAX2. *Arabidopsis edr1* mutants display spontaneous necrosis under drought suggesting that EDR1 acts to prevent drought-induced senescence (Tang et al., 2005). Assuming an ortholog of EDR1 can be identified in resurrection plants, an analysis of its expression and function during dehydration may be informative.

The interactions between cytokinin and ABA during water stress have revealed that cytokinin can inhibit the accumulation of ABA during water stress, and ABA can increase cytokinin accumulation during water stress (Pospíšilová et al., 2005). However, the reason for the accumulation of high levels of ABA and/or cytokinin in resurrection plants at the later stages of the desiccation process remains obscure. Studies have shown that high levels of growth-related gene transcripts accumulate at hydration levels well below 20% RWC (Le et al., 2007). This gene activity may occur in preparation for rapid growth following a rainfall event (Blomstedt et al., 2010).

## CONCLUSION

The ability to prevent initiation of drought-related senescence is a major component of the desiccation-tolerance program of resurrection plants. Mutational analysis in a number of non-resurrection plant species has revealed genetic mechanisms associated with promoting or progressing senescence. Current research has indicated that senescence can be activated in non-resurrection plants by a complex network of signal pathways that integrate metabolic signals with age-related information and environmental stress. As many defects in basic metabolic processes could potentially lead to premature cell death, identifying genes specifically involved in preventing senescence through mutational analysis is more problematic (Lim et al., 2007). In younger leaf tissues, resurrection plants can block the senescence signaling pathways and potentially provide a valuable resource for identifying these blocking mechanisms. Research in resurrection plants has indicated that desiccation-tolerance has not been conferred by the acquisition of genes unique to resurrection plants, but rather by alterations in the regulatory control of genes that are likely to be present in the genomes of most plants. Since drought-related leaf senescence is a survival strategy utilized by non-resurrection plants, the idea that these senescence blocking mechanisms currently operate in the some cells of reproductive or root tissues of resurrection plants may be worth exploring. As the input signaling into senescence is very diverse it seems likely that the leaves of resurrection plants harbor multiple mechanisms to repress drought-related senescence or alternatively may act at pivotal points of convergence. Recent senescence research indicates that ERD15, which integrates drought stress and ER stress signals to activate the NRP cell death pathway, represents one such convergence point and investigation of this pathway in resurrection plants may prove fruitful. ABA signaling may also feed into senescence via ERD15. Currently, the evidence that lysine-rich LEA proteins localized in various cellular compartments can attenuate ABA is not conclusive and warrants further investigation. Based on the precedence of the regulatory mechanisms controlling cell growth, induction of a particular cell death pathway may require both a positive activating signal and inactivation of a negative inhibitory signal. Recent results from Islam et al. (2013) suggests drought-, ABA-, and dark-induced senescence can be blocked by the activity of the *S. stapfianus* dehydration-responsive UGT SDG8i. SDG8i probably mediates this effect via repression of ORE9-mediated ubiquitination and degradation of senescence-inhibitory proteins. Further research into the key anti-senescence mechanisms associated with the desiccation-tolerance program may uncover valuable information on how these remarkable plants survive prolonged drought.

## Conflict of Interest Statement

The authors declare that the research was conducted in the absence of any commercial or financial relationships that could be construed as a potential conflict of interest.
